# WVSUD-PACT: a Primary-Care-Based Substance Use Disorder Team for Women Veterans

**DOI:** 10.1007/s11606-022-07577-3

**Published:** 2022-08-30

**Authors:** Sara Spinella, Nicole McCune, Rebecca McCarthy, Maria El-Tahch, Jennifer George, Mary Dorritie, Alyssa Ford, Kira Posteraro, Deborah DiNardo

**Affiliations:** 1 Department of Medicine, University of Pittsburgh School of Medicine, Pittsburgh, USA; 2 VA Pittsburgh Healthcare System, Pittsburgh, USA; 3 Waynesburg University, Waynesburg, USA; 4 Primary Care Mental Health Integration, VA Pittsburgh Healthcare System, Pittsburgh, USA

## INTRODUCTION

Women Veterans are the fastest growing demographic within the Veterans Health Administration (VHA).^1^ Substance use disorders (SUDs) are among the many conditions for which gender-specific considerations have implications for care delivery. Thirty-seven percent of women Veterans misuse alcohol and 16% have SUDs.^2^ SUDs are associated with key woman Veteran experiences, including combat and military sexual trauma.^2–4^ Increasing access to office-based SUD care has been an important goal in initiatives aimed at curbing SUD-related deaths.^5–7^ Doing so in other settings has been associated with improved acceptance of SUD care,^8,9^ lower costs,^10,11^ and increased uptake of preventive healthcare.^12^

Women with SUDs have complex needs in both primary care and SUD treatment domains. They have higher rates of unintended pregnancy and abnormal cervical cancer screens than those without SUDs.^13,14^ Among Veterans, women with SUDs are less likely to receive prescription contraception^15^ or medications for opioid use disorder (MOUD).^16^ Most women Veterans with at-risk alcohol use are not engaged in treatment.^17^ Women Veterans cite discomfort with mixed-gender programming and stigma surrounding mental health treatment.^18,19^ Experiences from within and outside VHA in providing integrated care for complex populations can be used to inform efforts to approach the care disparities and barriers as described for women Veterans with SUDs.

First, accumulating experience within VHA suggests that creation of woman-centered spaces is important to providing high-quality care to women Veterans, for both primary care and SUD care. VHA has emphasized access to designated women’s health providers (dWHPs), who have training in women’s health issues and women Veteran experiences, for provision of gender-specific primary care.^20^ There are higher rates of satisfaction, cervical screening, and breast cancer screening among those seen by dWHPs.^21–23^ Similarly, increased engagement with SUD treatment within VHA has been associated with centers that offer women-only programming.^18^ Half of women Veterans in SUD treatment attend women-only groups when available.^24^ Outside VHA, availability of gender-specific services has been positively associated with receptivity to receiving SUD care, retention rates, and treatment outcomes.^25–27^

Secondly, VHA has extensive experience with the benefits of interdisciplinary team-based care delivery through Patient Aligned Care Teams (PACTs). PACTs are comprised of a primary care provider, registered nurse (RN), medical assistant (MA), licensed practical nurse (LPN), and administrative clerk, who work to manage chronic disease through patient-centered communication and care coordination.^27^ Integrated mental health services and clinical pharmacy specialists (CPSs) are imbedded in the PACT model. Women Veterans have an increased need for time-intensive care coordination from the PACT team^28^ and panel sizes are therefore reduced.^29^ Previously described PACT-based care tailored to high-needs Veterans highlights the importance of integrated comprehensive services, continuous staff training, and intensive care management.^30,31^

Finally, existing evidence suggests that coordination across the care continuum is important for SUD care. Several models of MOUD provision have been described.^7^ In hub and spoke care, stable patients are managed locally while complex patients are referred to centralized addiction services.^5,6^ In “shared care” models, addiction psychiatry initiates MOUD while primary care maintains it.^32^ There are also benefits to linking inpatients with SUDs to outpatient treatment; those who initiate buprenorphine with outpatient MOUD follow-up are less likely to relapse than those who receive detoxification alone.^33^

Based on the existing literature as described and with a goal of addressing the unique barriers to care experienced by women Veterans with SUDs, we used the interdisciplinary PACT structure to extend gender-sensitive primary care, SUD pharmacotherapy, and psychologic support to women Veterans with SUDs. We present this model for a women Veterans SUD PACT (WVSUD-PACT) by describing: (1) characteristics and early outcomes of enrollees, (2) an evidence-based framework for program development, and (3) future directions.

## SETTING AND PARTICIPANTS

In 2020, VHA Women’s Health Services supported a pilot WVSUD-PACT for women Veterans with SUDs through the Women’s Health Innovation and Staffing Enhancements (WH-ISE) grant. The WVSUD-PACT is housed within a comprehensive women’s health center in Pittsburgh and as of May 2021 has served 25 Veterans.

## PROGRAM DESCRIPTION

**Fig. 1.**
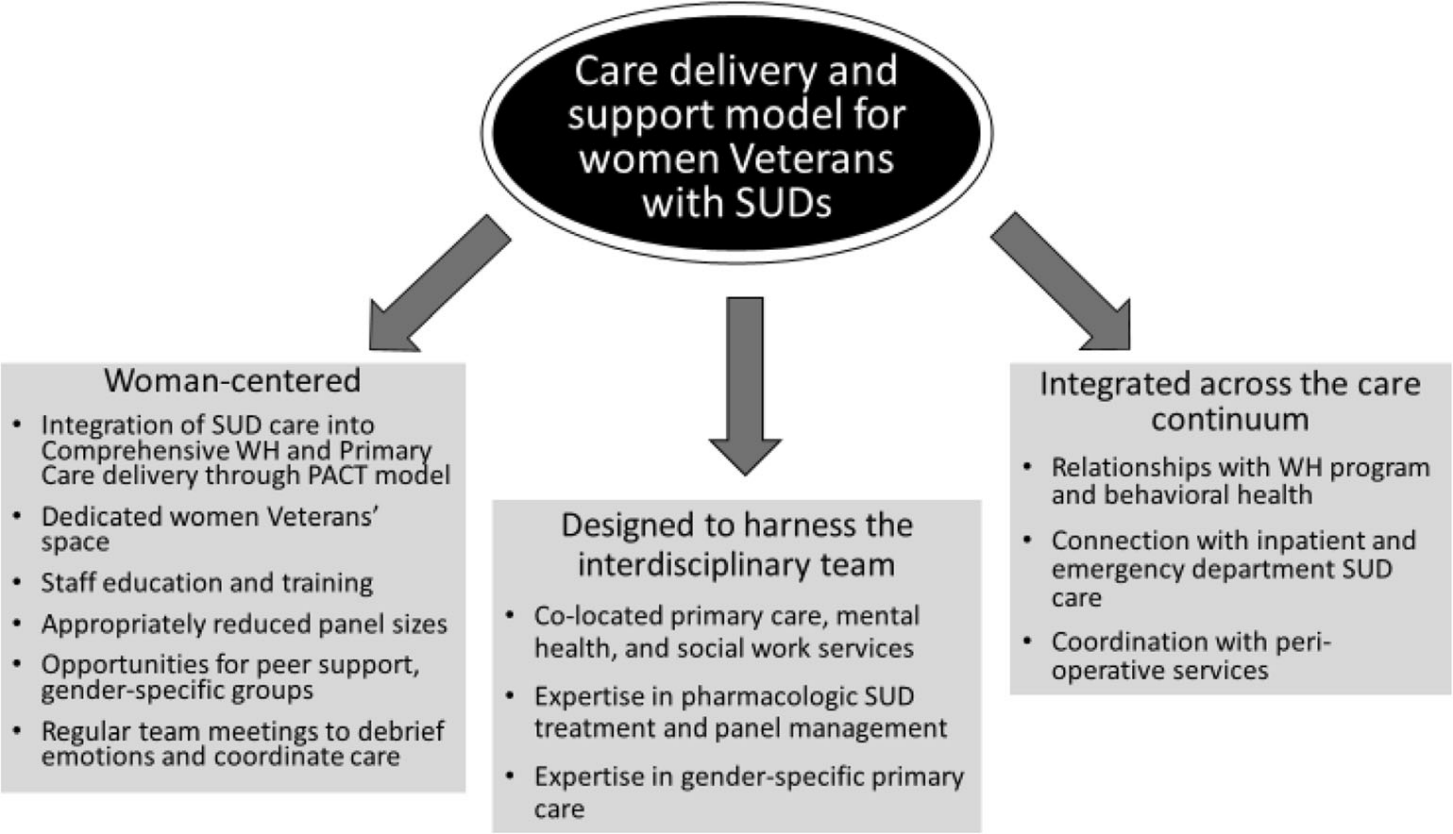
Innovative model for delivery of health care to women Veterans with SUDs. The optimal care delivery is: (a) trauma-informed and woman-centered, (b) designed to harness the power of an interdisciplinary team with complimentary skills, and (c) integrated across the care continuum, from inpatient to specialty SUD care to management of stable recovery.

We highlight three key features of our model (Fig. 1).

### Woman-Centered Care

As with all women’s health PACT teams, WVSUD-PACT members have experience working with women Veterans. The team includes a dWHP, LPN, RN, social worker, CPS, psychologist, and administrative clerk. Because SUDs occur in the context of Veterans’ gendered experiences, our psychologist has expertise in both SUDs and gender-specific concerns like trauma, intimate partner violence (IPV), eating disorders, and reproductive mental health; this content is integrated into therapeutic plans. Consistent with standards for comprehensive women’s health centers, the clinic has a dedicated waiting room, bathroom, and exam room.^29^ We are also developing women-only therapy groups.

Though panel size for dWHPs is smaller than for those who see male Veterans,^29^ we have further reduced panel size by 50% to enable close follow-up. Additionally, the team meets monthly to debrief emotions and develop SUD- and gender-specific skills, similar to the continuous staff education described elsewhere.^30,31^

### Harnessing the Interdisciplinary Team

Our model depends on the expertise of a multidisciplinary team. Our integrated psychologist is available for both scheduled and same-day meetings with Veterans to assess mental health crises, provide motivational interviewing and relapse prevention, promote health behavior change, and serve as a liaison to mental health services. Our CPS monitors patients on medications for alcohol (MAUD) and opiate use disorder (MOUD) for adherence, side effects, and efficacy, and increases the availability of the dWHP.

### Involvement Across the Care Continuum

Key to our model is a close relationship between inpatient SUD care and the WVSUD-PACT. This connection enhances provision of gender-informed inpatient SUD care and provides opportunities to establish rapport and arrange follow-up. Additionally, women Veterans with SUDs often have complex medical and psychiatric needs. Protected non-clinical time for the team leader enables her to cultivate interdisciplinary relationships and coordinate care; we arrange meetings between our team, specialty addiction services, pain management, anesthesia, surgical services, and palliative care for patients with complex pain diagnoses or peri-operative pain concerns.

Our model also relies on deliberate relationship-building and care coordination with specialty addiction services. The WVSUD-PACT facilitates referrals for patients who are struggling with primary-care-based SUD treatment or have comorbid psychiatric conditions outside the scope of primary care. Through this mechanism, Veterans can receive long-term individual therapy, high-frequency group meetings, residential treatment, or methadone treatment. We continue to provide SUD-informed and gender-specific primary care throughout periods of specialty SUD and/or mental healthcare and are available to resume primary-care-based SUD treatment when appropriate.

## PROGRAM EVALUATION

This pilot program was launched in February 2021. By May 2021, 25 of 36 women referred to the WVSUD-PACT had joined. Early review of WVSUD-PACT patient characteristics suggests that our care model is filling an important gap in care; many Veterans had not received recent SUD and/or primary care (Table 1). Additionally, the WVSUD-PACT is serving a high-risk patient population. The Care Assessment Needs (CAN) score is an outpatient risk stratification tool ranging from 0 to 99 used to calculate the relative risk of death or readmission based on data about sociodemographics, comorbidities, vitals, healthcare utilization, medications, and labs. The average CAN score of enrolled WVSUD-PACT Veterans is 82, compared with an average CAN score of 60 for general women in dWHP panels at our site. Chronic pain is among the most common comorbidities in our panel.

**Table 1:** Characteristics of Patient Panel in Pilot Women’s Health SUD Clinic

**Average age**	50
Average CAN* score	86.5
**Substance of concern**	
*Alcohol*	12 (48%)
*Benzodiazepine*	1 (5%)
*Cocaine*	14 (56%)
*Opioids*	13 (52%)
**Comorbidities**	
*Pain diagnosis*	18 (72%)
*Current tobacco use*	18 (72%)
*MST†*	13 (52%)
*Past year IPV‡*	3 (12%)
*Heart failure/stroke*	6 (24%)
*Diabetes mellitus*	5 (20%)
*Depression*	16 (64%)
*Cannabis use in the last 2 years*	12 (48%)
**No engagement in the past 2 years with…**	
*dWHP§*	4 (16%)
*Addiction treatment*	9 (36%)
*Behavioral health*	8 (32%)
**Pharmacotherapy**	
*Total prescribed*	20 (80%)
*Initiated or re-initiated by clinic*	6 (24%)

*CAN, Care Assessment Needs. For comparison, the average CAN score for all women in dWHP panels at our site of 60. *†* MST, military sexual trauma. *‡ IPV*, intimate partner violence. *§*, designated women’s health provider

Early review of our WVSUD-PACT patients also demonstrates a variety of substances of concern, with many patients struggling with more than one substance. Cannabis use was common within the WVSUD-PACT but was never identified as a substance of concern by referring providers or patients.

Though long-term patient outcome data are not available, nearly 75% of enrolled patients are prescribed pharmacotherapy for their SUD and 22% were started on medications through WVSUD-PACT. Since enrolling in this program, 4 of 7 women overdue for cervical cancer screening have completed testing and 2 of 7 women overdue for colorectal cancer screening have completed testing.

## DISCUSSION

The WVSUD-PACT was developed to enhance both SUD and primary care delivery to women Veterans through a multidisciplinary team with expertise in women’s health and SUDs. Pilot program data suggests this model can increase engagement in preventive healthcare and access to pharmacotherapy for women Veterans with SUDs. This pilot was structured around three key features: woman-centered care, the interdisciplinary team, and involvement across the care continuum.

While our program shares features with other primary care models for the treatment of SUDs,^7^ WV-SUD PACT is unique in its focus on woman-centered care. Gender-conscious SUD care foregrounds issues that may put women at risk of relapse, including caregiving responsibilities, fertility challenges, past trauma, IPV, and high rates of psychiatric comorbidities.^34^ These same issues may impact Veterans’ experiences with medical care; our physical space and the women’s health background of our team enable us to meet the gendered needs of our population.

An interdisciplinary approach and co-location of primary and specialty services is important in care models for other vulnerable patient populations.^30,31,35^ Many models integrate SUD counselors and mental health professionals into primary care^36^, and VHA is a leader in mental health integration.^37^ We built on this work by including a psychologist with specific experience in women’s health and SUD treatment to provide short-term individual therapy and to develop groups. Our model also builds on VHA experience utilizing CPSs to improve outcomes for chronic diseases.^38^ Outside VHA, pharmacist-led interventions have been shown to improve adherence to psychiatric medications^39^ and to increase MAUD within addiction treatment programs.^40^ Within VHA, efforts to expand CPS practice to include management of SUDs have had encouraging results.^41^ We capitalize on CPS collaboration to maximize the benefits of MAUD/MOUD in this clinic. CPSs with expertise in MAUD/MOUD may be particularly useful should this model be adapted by dWHPs without extensive training in addiction medicine.

Finally, we work with Veterans throughout the course of their recovery in multiple settings. Evidence supports initiation of long-term MOUD during inpatient stays.^33^ The WVSUD-PACT seeks out hospitalized women Veterans with SUDs to initiate treatment, with an added benefit of inpatient and outpatient continuity. Like other models for office-based SUD care, including “shared care”^32^ and “hub and spoke” models,^5,6^ a relationship with specialty addiction treatment for care of complex cases is crucial. However, we adapted these models to address the specific needs of women Veterans by offering MAUD/MOUD initiation for Veterans who may feel uncomfortable in mixed-gender settings and by providing SUD-sensitive primary care even when a patient is receiving specialty SUD care.

Though evaluation of this early pilot is limited, two lessons from our experiences thus far stand out. First, recruitment to this PACT can be challenging. Some Veterans worry about stigma within the healthcare system if they belong to a PACT for people with SUDs, while others do not want to switch to a new dWHP. Concerns about stigma may be partially mitigated by removing the SUD-identifiers from the PACT name within the electronic medical record. Expanding WVSUD-PACT services to include a consultative role for some women may increase access to MOUD/MAUD and integrated mental health services for those who wish to continue with their prior dWHPs but are reluctant to receive care through behavioral health.

Secondly, many Veterans in our WVSUD-PACT struggle with chronic pain, which may exacerbate SUDs.^42^ Women Veterans without SUDs have described experiences of stigma related to chronic pain and the desire for woman-centered pain programs.^43^ Pain management is often the focus of visits with our WV-SUD PACT and is sometimes a barrier to discussion of MOUD/MAUD or involvement in SUD therapy. Management of the pain/addiction interface will be crucial to the success of the program going forward. Our psychologist is completing training in cognitive behavioral therapy for chronic pain and our CPS is expanding her role to include management of chronic pain.

As we grow this program, we will assess features that promote long-term engagement with care and evaluate women’s health and SUD outcomes. However, our early results suggest we are already reaching a vulnerable segment of the growing women Veteran population.
